# A Case of Heroin Induced Sensorineural Hearing Loss

**DOI:** 10.1155/2014/962759

**Published:** 2014-01-06

**Authors:** Ricardo Mario Aulet, Daniel Flis, Jonathan Sillman

**Affiliations:** ^1^Tufts University School of Medicine, 145 Harrison Avenue, Boston, MA 02111, USA; ^2^Department of Otolaryngology-Head and Neck Surgery, Tufts Medical Center, 800 Washington Street, Boston, MA 02111, USA

## Abstract

A case of a 31-year-old male who developed profound sensorineural hearing loss following a heroin overdose is presented. The patient subsequently had a full recovery of his hearing. Other cases of this rare phenomenon are reviewed and management options are discussed.

## 1. Introduction

There are only a few cases of heroin overdose associated hearing loss reported in the literature [1–5]. More common complications of heroin overdose include physical injury from falling or loss of consciousness, vomiting, and chest infections [6]. Well-known neurologic complications include peripheral neuropathy, temporary limb paralysis, transverse myelitis, seizures and stroke [6–8]. Recovery of hearing was seen in all but one of five cases of heroin overdose associated hearing loss. We present a patient who had SNHL with concomitant rhabdomyolysis after intravenous heroin overdose and provide a review of the literature.

## 2. Case Report

A 31-year-old male with a history of opiate abuse, including previous overdoses, presented to the emergency department of an outside hospital after being found unresponsive by his family. Heroin was found at his bedside by his family. On presentation to the emergency department, he had pinpoint pupils, was hypotensive, and was difficult to arouse. He was given Naloxone and resuscitated with intravenous fluids.

**Figure 1:**
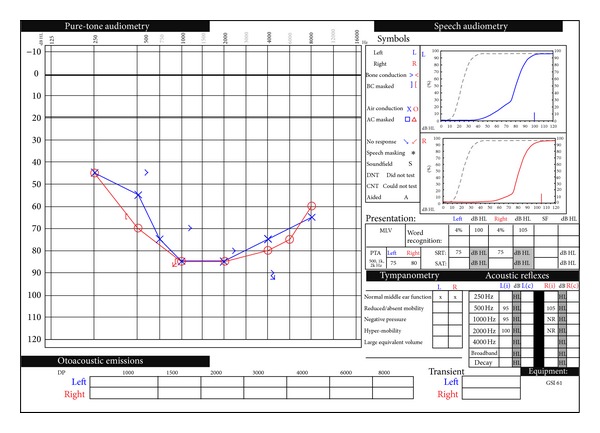
Initial pure-tone audiogram showing bilateral moderate to severe sensorineural hearing loss and speech audiometry showing poor word discrimination bilaterally.

As his mental status improved, he complained of new onset bilateral hearing loss, abdominal pain, and nausea. His urine toxicology was positive for opiates and negative for all other substances. His blood chemistry was consistent with rhabdomyolysis with a creatinine of 1.4, lactate of 3.2, CPK of 2,397, and potassium of 6.0. He was admitted to the critical care unit for further management. A CT Head was performed that showed infratemporal air but was otherwise normal with no evidence of infarct. At the outside hospital, he was also given one dose of IV solumedrol 120 mg. He was stabilized and transferred to our institution for further evaluation. A formal audiogram was performed, which demonstrated moderate-to-severe bilateral sensorineural hearing loss. On his audiogram, he was noted to have very poor word discrimination bilaterally (Figure 1). His tympanograms were normal. An MRI/MRA was performed and showed no other abnormalities. He started on a one-week course of 60 mg prednisone daily and then a one-week tapered course. His rhabdomyolysis resolved with intravenous fluids and he was discharged home on hospital day 4.

**Figure 2:**
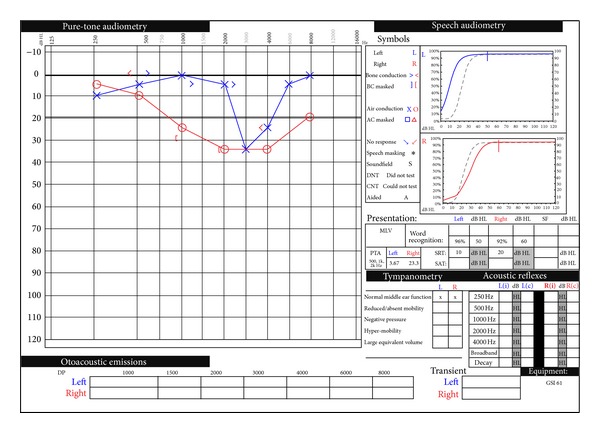
Follow-up pure-tone audiogram showing mild, mixed, and mid range hearing loss on the right and mild sensorineural hearing loss at 3000 hz on the left improved from prior study. Speech audiometry showing improved speech discrimination over prior study.

One week later, he was seen as an outpatient. He reported significant improvement in his hearing, noting greater improvement on the left than, the right. He noted some tinnitus and fullness in his right ear but denied any other otologic symptoms. His audiogram demonstrated mild, mixed, and mid frequency on the right and mild sensorineural hearing loss on the left at 3000 hz (Figure 2). His word discrimination was normal bilaterally. He was lost to follow-up after this visit.

## 3. Discussion

Sudden sensorineural hearing loss after intravenous heroin overdose has been described in five previous cases [1–5]. In all cases, they described the overdose occurring during a relapse after a period of abstinence [1–4]. The patients fully recovered between three days and three weeks in three out of five of the reports [1–3]. Two of the cases were described using prednisone. Recovery occurred in only one of the two patients treated with prednisone [3, 5]. Vasodilators such as xanitol, nicotinate, and pentoxyfilin were used in three of the cases, with two patients fully recovering [2–4].

The mechanism of heroin overdose associated hearing loss is not well understood. Several theories have been proposed to explain this phenomenon.

Like the patient in our case, many of these patients are hypotensive on presentation. Previous studies have shown a possible link between hypotension and sensorineural hearing loss [9, 10]. However, these reports found hypotension associated hearing loss to occur in the lower range frequencies, which is different than the patient in our case. It is suggested that low frequency loss is more likely to result with hypotension related hearing loss because the apex of the cochlea receives terminal blood supply and is, therefore, more vulnerable to hypotension [10].

Studies in rats and guinea pigs have found the presence of mu, kappa, and delta opioid receptors on the cochlea [11, 12]. Activation of the kappa receptor on cochlear hair cells decreases afferent activity [13]. In the setting of acute opioid overdose, overstimulation of these kappa receptors and decreased afferent activity in the cochlea could be a possible explanation for the hearing loss that occurs. In the few reported patients that failed to recover from opioid and acetaminophen induced sensorineural hearing loss, cochlear implants have been successfully used, which suggests a possible cochlear origin of the hearing loss [14].

Another possible explanation is that the heroin was mixed with an ototoxic drug. Heroin has been reported to be mixed with quinine. Quinine toxicity is a well-known cause of reversible high frequency hearing loss. There have been cases of quinine toxicity effects being seen in patients after injection of what they thought was heroin [15, 16].

At this time, the complete explanation of opiate induced hearing loss is not known. Although steroids have been used for treatment in other cases, there is not sufficient evidence supporting their use. Fortunately in our case and in most of the other cases reported in the literature, the hearing loss was reversible and patients recovered. Future research examining the effects of opiates on the functions of the inner ear is needed to better understand the physiological process and response to these drugs.
